# Development of Personalised Immediate-Release Gel-Based Formulations Using Semi-Solid Extrusion

**DOI:** 10.3390/gels10100665

**Published:** 2024-10-17

**Authors:** Morenikeji Aina, Fabien Baillon, Romain Sescousse, Noelia M. Sanchez-Ballester, Sylvie Begu, Ian Soulairol, Martial Sauceau

**Affiliations:** 1 RAPSODEE, IMT Mines Albi, CNRS, University of Toulouse, 81013 Albi, France; 2 ICGM, University of Montpellier, CNRS, ENSCM, 34293 Montpellier, France; 3 Department of Pharmacy, Nîmes University Hospital, 30029 Nîmes, France

**Keywords:** personalised medicine, caffeine, semi-solid extrusion, immediate release, stability, forced degradation, batch variability, agar, hydroxypropyl methylcellulose (HPMC)

## Abstract

Precision in dosing is crucial for optimizing therapeutic outcomes and preventing overdosing, especially in preterm infants. Traditional manual adjustments to adapt the dose often lead to inaccuracies, contamination risks, and reduced precision. To overcome these challenges, semi-solid extrusion 3D printing was used to create personalised gel-based caffeine dosage forms. The hydrogels, made from agar and hydroxypropyl methylcellulose, demonstrated excellent rheological properties, ensuring uniform extrusion and accurate shape retention during and after printing. This gel formulation allowed for precise adjustments of caffeine volume and content tailored to a neonate weighing 1.36 kg, achieving a recovery of 103.46%, well within acceptable limits. Additionally, three production batches confirmed the process’s reproducibility with minimal variability. Forced degradation studies showed that both pure caffeine and caffeine in the gel matrix exhibited similar stability profiles, confirming the drug’s chemical integrity. The printed gel dosage forms also displayed immediate-release characteristics, with over 80% of caffeine released within 45 min, highlighting their suitability for rapid therapeutic action. These findings emphasise the potential of SSE 3DP and gel-based formulations to produce personalised drug delivery systems with high precision, reproducibility, and reliability.

## 1. Introduction

Caffeine (1,3,7-trimethylxanthine) is a psychostimulant purine-like alkaloid that is readily soluble in water [1,2]. In infants, caffeine and other methylxanthines act on the central and peripheral receptors, stimulating the medullary respiratory centre thus, influencing wakefulness and sleep [3,4]. Compared to other methylxanthines such as aminophylline and theophylline, caffeine has a wider therapeutic index and longer half-life. Caffeine can, therefore, be administered once a day for the treatment of apnoea of prematurity (AOP) among neonates [4,5]. In the neonatal intensive care units (NICU), the infants are typically administered an intravenous loading dose of 20 mg/kg of caffeine citrate (10 mg/kg of caffeine base) followed by a daily maintenance oral dose of 5–10 mg/kg of caffeine citrate (2.5–5 mg/kg of caffeine base) [4,5,6]. As the volume to be administered needs to be adjusted to the weight of the infant, dosing errors could occur due to human errors, low accuracy, and/or contamination [7].

Three-dimensional (3D) printing is based on the layer-by-layer building of an object (model) using computer-aided design (CAD) [8]. This model can be adjusted to meet the user’s requirements, 3D printing techniques thus, can be employed to produce flexible dosage forms in terms of dosage, geometry, drug release kinetics, and composition [9]. The main 3D printing techniques employed for pharmaceutical applications include fused deposition modelling (FDM), semi-solid extrusion technology (SSE), direct powder extrusion (DPE), inkjet printing (IP), stereolithography (SLA), and selective laser sintering (SLS) [10]. SSE has however, gained popularity due to its application in obtaining solid dosage forms with specific characteristics, such as chewable, gummy, fast-dissolving, or gastro-floating tablets, polypills, and oral films, among others [11]. SSE also requires low or no heat, thereby reducing the risk of drug degradation, and the possibility of using a wider range of excipients as feedstock materials to obtain gels or pastes for the 3D printing process (excipients such as natural or synthetic polymers, lipids, and food additives) [11,12].

Since caffeine is a small molecule with a molecular weight (MW) of 197.2 g/mol, it can pass through the buccal epithelium by passive diffusion [13,14]. Thus, 3D-printed oral dosage forms of caffeine are good alternatives to the manual volume adjustment currently done for its administration to neonates with AOP. Since, as demonstrated in previous studies [15,16,17], one can accurately estimate the required dose based on the weight or volume of the printed forms or preset models. Moreover, the printed oral dosage forms containing the precise dose needed can either be dispersed in milk or water before administration [18,19]. In addition, Krueger et al. [20] reported that the accuracy and precision of 3D-printed forms exceed those obtained using the traditional method of manual subdivision of commercially available tablets. However, Johannesson et al. [15] observed that changes in rheological properties will influence the amount of formulation extruded during 3D printing and consequently, results in varied drug content across batches.

Hydrogels are versatile materials widely utilized for their ability to retain large amounts of water, making them suitable for various biomedical and pharmaceutical applications, including drug delivery, wound healing, and tissue engineering [21,22]. Numerous studies have explored different hydrogel systems, each focusing on the unique properties of their base materials. For example, natural polymers such as alginate and gelatin have been used to develop biocompatible hydrogels with optimized degradation behavior and swelling capacity [23]. Meanwhile, synthetic polymers like polyethylene glycol (PEG) and polycaprolactone (PCL) have been combined to enhance both mechanical stability and biodegradability [24]. However, balancing mechanical properties with biocompatibility remains a key challenge in these studies.

In this study, agar, a natural polysaccharide, and hydroxypropyl methylcellulose (HPMC), a semi-synthetic polymer, were selected as hydrogel-based polymers to address these limitations by leveraging their complementary properties. Agar is known for its excellent gelling properties even at low concentrations, providing structural integrity and mechanical stability, making it ideal for soft yet robust matrices [25,26]. HPMC, with its film-forming and swelling capabilities, improves the extrudability and handling of gel-based formulations [27,28]. Both polymers are widely used in pharmaceutical and food products, which makes them particularly suitable for neonatal applications. Their non-toxic, biocompatible nature minimizes the risk of adverse effects in preterm infants, as demonstrated in multiple studies [18,29,30,31]. Additionally, these hydrogels are easy to fabricate, allowing for the incorporation of various therapeutic agents and making them ideal for point-of-care applications tailored to the specific needs of vulnerable populations like preterm infants.

As part of a drug development program, the ICH Q1A(R2) guideline mentions that stability studies are important for establishing storage conditions, determining the re-test period for the drug substance, and/or defining a shelf life for the drug product. However, stability studies can be time-intensive, typically requiring 6 months for accelerated and 12 months for long-term assessments. As a faster alternative, forced degradation studies are used to estimate pharmaceutical product stability [32]. During a forced degradation study, drug products and substances undergo degradation under conditions more severe than those used in accelerated degradation tests, resulting in the formation of degradation byproducts [32]. The presence or absence of such degradants peak in the chromatogram of stored samples indicates the chemical degradation or potential stability of the drug product in the storage condition, respectively. An extent of 5–20% degradation of the active pharmaceutical ingredient (API) is generally considered acceptable [33,34] since over-stressing a sample could lead to the formation of secondary degradation products that are not observable in formal shelf-life stability studies [32,35].

In addition to chemical stability concerns, gel-based formulations introduce potential physical variabilities. One key source of variability during storage or across batches is the rheological properties of the gel, which can affect the printability of the dosage form. Variability in these properties may result in inconsistent layer deposition, leading to variations in drug content and dosage uniformity. Furthermore, subtle variations in gel composition or environmental factors (such as temperature or humidity) can impact the physical state of the gel, potentially affecting the drug release profile and stability of the dosage form [15,36]. Achieving uniform drug distribution within the gel matrix can also be challenging, which may result in inconsistent dosages and therapeutic outcomes [37,38]. These factors highlight the need for rigorous testing of both chemical stability and physical properties to ensure the quality and reproducibility of 3D-printed gel-based dosage forms.

Therefore, in this study, the potential variabilities associated with personalised gel-based dosage form was evaluated using an agar-HPMC-based matrix loaded with caffeine. In addition, the stability of the gel-based matrix was investigated for instances where an excess of the gel feedstock or dosage form is produced. However, due to the absence of antimicrobial agents and the high moisture content of the current formulation, the stability study was limited to three days. As an alternative to accelerated stability tests, forced degradation studies were also performed. Ultimately, the results presented in this study aim to describe the predictive modelling, stability characteristics, and potential variabilities that may occur in 3D printed gel-based dosage forms.

## 2. Results and Discussion

### 2.1. Evaluation of Quality Control Attributes

**Figure 1:**
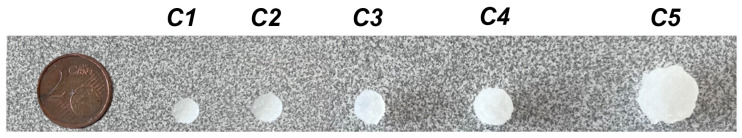
Printed forms at 100% infill density. C1, C2, C3, C4 and C5 represent cylindrical objects of different dimensions as described in Table 1.

An image of the printed forms is shown in Figure 1. This image provides a visual representation of the forms manufactured using the method described in Section 4.3. Upon examination, it was observed that the surface of the printed forms exhibited a gummy appearance, indicative of their ability to serve as soft and chewable dosage forms. In addition, the forms generally conformed to the intended cylindrical shape. However, it was observed that the printed forms suffered from poor resolution, primarily due to the diameter of the printing nozzle employed. To address this limitation and enhance resolution, consideration could be given to utilizing a print head with a smaller nozzle diameter. This adjustment could yield objects finer details and smoother surfaces. Nonetheless, despite the observed resolution issues, the current morphology of the printed forms was deemed acceptable for further analysis.

As illustrated in Table 1, compared with the pre-designed size, the dimensions of all the printed forms were significantly larger (*p* < 0.05). The inability to adjust the width of the printed filament can be attributed to the unique properties of the gels, which contain both liquid and solid components [39]. When extruded from the syringe, the gels exhibit behaviour similar to that of a liquid. Consequently, any attempt to reduce the line width is ineffective, as the material invariably adopts the width of the nozzle [40]. Considering this, it would mean that trying to fit an infill into the already enlarged width would result in more material spreading to comply with the designed object. Indeed, it was observed that the higher the infill density, the larger the deviation from the designed size. Despite this, the dimensions of the printed object were repeatable (RSD < 5%).

Since the volume of the forms was greater than the designed forms, the mass was inadvertently greater than desired. Nevertheless, the Ph. Eur. requirements for forms of a mass over 250 mg stipulate that no more than two forms in a batch of 20 should deviate from the average mass by more than 5%, which all the printed forms complied with, as shown in Table 2. Subsequently, the drug content of 10 printed forms of each dimension was analysed by UV to determine the average caffeine content in each dosage unit. The forms yielded mean doses and their acceptance value for a label claim of the nearest tenth decimal point was less than 1.5 (shown in Table 2). The dosage units, thus complied with the Ph Eur.

In addition, as shown in Table 2, all samples except for the largest dosing unit (C5) disintegrated within 180 s. This rapid disintegration suggests that these units are likely to dissolve more quickly, which could potentially enhance the bioavailability of the drug [41]. However, the largest dosing unit (*C5*) exhibited a prolonged disintegration time of 345 ± 9 s. As shown in Table 2, this extended disintegration time may be attributed to its lower surface area to volume ratio (SA/V), which can hinder water penetration and prolong the disintegration time [42]. Additionally, the higher HPMC content in the larger dosing units may also contribute to the slower disintegration. Initially, the HPMC in all dosing units forms an outer gel layer upon contact with water. This gel layer creates a barrier that could slow down water penetration and disintegration [43]. Therefore, dosing units with more HPMC content would form a larger and more substantial gel layer, resulting in slower water penetration and a higher disintegration time.

**Figure 2:**
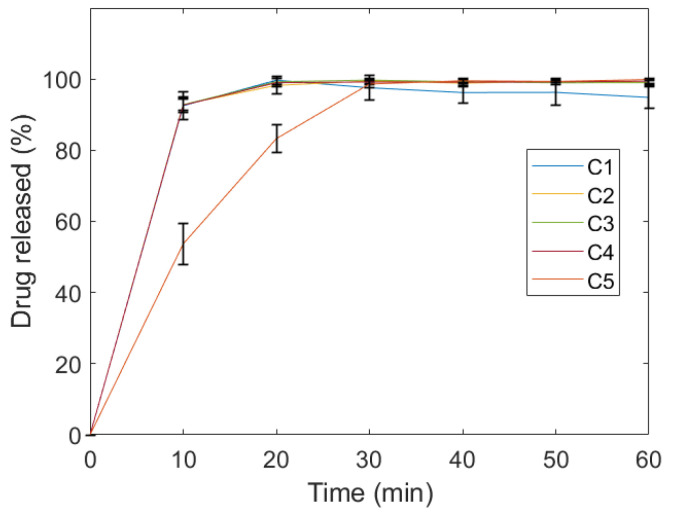
Dissolution curve of the cylindrical models. C1, C2, C3, C4 and C5 represent cylindrical objects of different dimensions as described in Table 1.

This observation became more apparent when the dissolution of the dosing units was compared. As shown in Figure 2, while all other dosing units achieved over 80% drug release within 10 min, sample *C5* only reached 53% at the same time point. Despite the decreasing SA/V ratios observed in the other dosing units, sample *C5* exhibited both the lowest SA/V ratio and the longest disintegration time (Table 2), resulting in the most pronounced impact on its drug dissolution behaviour. Indeed, the reduced surface area available for interaction with the dissolution medium, coupled with the prolonged time required for disintegration, led to a significantly slower drug release compared to the other units. Similar findings regarding slower drug release associated with lower SA/V ratios have been documented in several studies [44,45,46]. Yet, *C5* can be considered an immediate release dosage form according to Ph. Eur. 5.17.1, 51701 (01/2023), as they released more than 80% of caffeine in less than 45 min. Moreover, with an $f_{2}$-value of over 90 between C5 and other samples, this dosing unit can be considered similar regarding the release of caffeine. It is, however, interesting to note that extended-release dosage forms may also be fabricated using the same formulation by simply increasing the dimensions of the geometric model or reducing the SA/V.

### 2.2. Personalisation of Dosage Form

**Figure 3:**
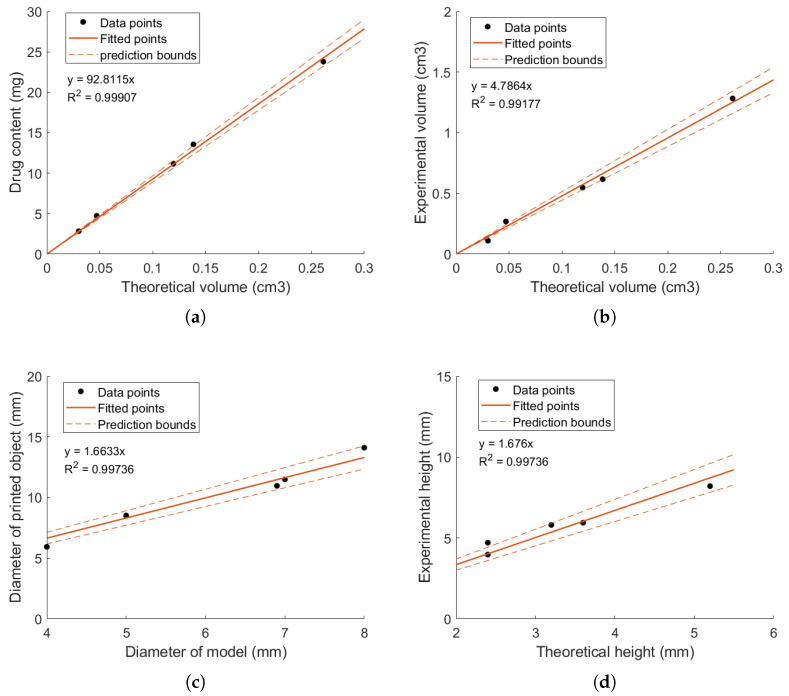
Linear relationships between theoretical volume and drug content (**a**), theoretical volume and experimental volume (**b**), theoretical diameter and experimental diameter (**c**), and theoretical height and experimental height (**d**). Prediction bounds are model estimates at 95% confidence intervals.

As shown in Figure 3a, a strong linear correlation between the drug content and the theoretical volume was achieved, as evidenced by an $R^{2}$ value of 0.999. Despite this high correlation, it was observed that the dimensions of the printed objects deviated significantly from the model’s dimensions. To address this discrepancy, a linear regression equation was developed to predict these variations, as presented in Figure 3b. Both linear regressions demonstrated high reliability, with $R^{2}$ values exceeding 0.98, indicating their suitability for use in quality assurance [47]. Using the linear regression equation from Figure 3a, the theoretical volume required to print a dosing unit with a specific caffeine content was determined. Based on this estimated theoretical volume, the height, *h*, of the model to print was calculated for a desired diameter, while taking into account the layer height used in the printing process (Figure A4). Subsequently, the diameter and height of the printed objects were estimated using the regression equation presented in Figure 3c and Figure 3d, respectively.

A screen capture of the process, along with the proposed dimensions for a dosage unit containing 3.4 mg of caffeine, is shown in Figure A4. According to the screen capture, a cylindrical dosing unit containing 3.4 mg of caffeine should be printed with a diameter of 4 mm and a height of 2.8 mm. The script further estimated the printed object’s dimensions to be 6.7 mm in diameter and 4.7 mm in height. However, the actual printed model measured 5.9 mm in diameter and 4.7 mm in height, resulting in percentage errors of −15.3% for the diameter and −0.9% for the height. Despite these dimensional discrepancies, the percentage of caffeine recovered from the printed object was 103.5%, and the acceptance value calculated for 10 printed objects was well within the acceptable range (AV = 2.1). Moreover, the predicted drug content within the printed object ranged between 3.3 and 3.5 mg at a 95% confidence interval, underscoring the regression equation’s precision and reliability. Overall, these findings indicate that the regression equations were effective in minimizing the trial-and-error approach traditionally required to determine the appropriate dimensions for a given dose. Integrating such data collection into the validation process of a 3D printer could significantly enhance the accuracy and reliability of printed pharmaceutical products. This approach not only ensures that the finished drug product meets its designed attributes but also contributes to more efficient and consistent production of personalised medicine.

### 2.3. Identification of Variability

**Figure 4:**
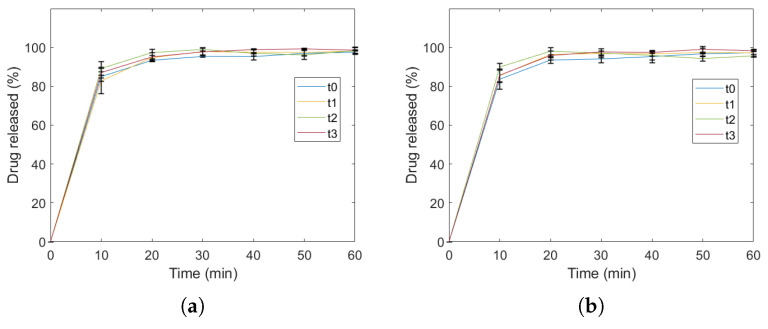
Dissolution of the proposed cylindrical models (diameter: 4 mm and height 2.8 mm) across 3 batches and at four time points, for samples stored at 25 °C and 60% RH (**a**) and samples stored in the refrigerator (**b**). The time points investigated were immediately after printing (t0), after 1 (t1), 2 (t2) and 3 (t3) days.

From Figure 4, all the printed forms across the three batches, storage length and storage conditions, showed immediate release drug profile ($f_{2}$-value > 90). Therefore, no analysis of variance (ANOVA) was conducted.

**Figure 5:**
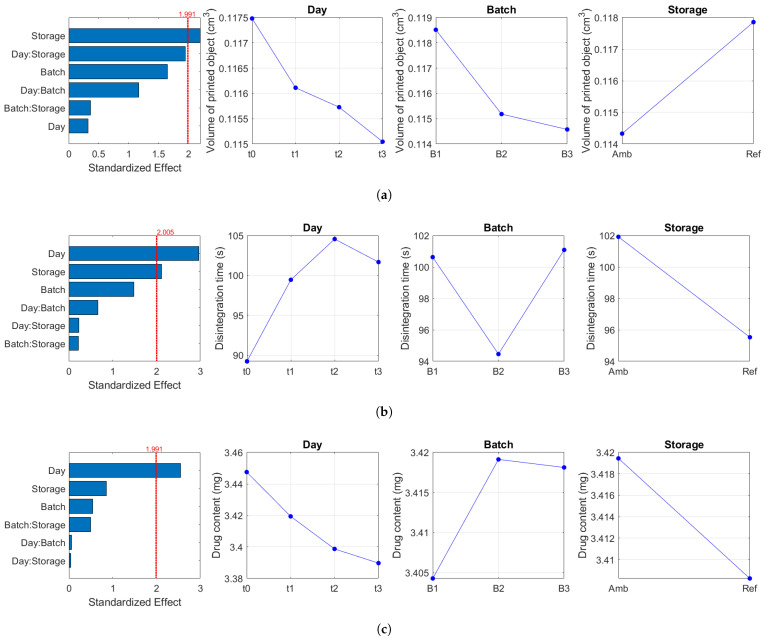
Standardised effects of the factors on the volume of the printed object (**a**), disintegration time (**b**) and drug content (**c**). The time points investigated were immediately after printing (t0), after 1 (t1), 2 (t2) and 3 (t3) days. The dotted red line corresponds to the critical value; effects exceeding this threshold are considered statistically significant.

For the other attributes, selected results of the ANOVA is presented in Figure 5. In such figures, significant factors are identified as bars crossing the dotted red line, corresponding to the critical value. For example, as illustrated in Figure 5a, the type of storage condition had a statistically significant effect on the volume of the printed objects (p=0.031). This observation is attributed to the hydrophilic and/or hygroscopic nature of gel-forming excipients used in the formulation. These excipients have a strong affinity for water and readily absorb moisture from their surroundings. In the humid and cooler environment typical of a refrigerator [48], these polymer chains absorb water, causing them to swell and expand [49,50]. Consequently, the printed objects stored in the refrigerator (*Ref*) exhibited higher volumes compared to those stored at 25 °C and 60% RH (*Amb*). Moreover, the increased swelling of the matrix due to water uptake may result in polymer chains with greater mobility, leading to a higher phase angle or more fluid-like properties [51,52]. This behaviour was observed in the rheological analysis of feedstocks stored in the refrigerator (Figure A5a).

As illustrated in Figure 5b, the length of storage time resulted in the highest statistically significant difference (p=0.004). This effect is likely due to changes in moisture content over time [53,54], which made it difficult for water to penetrate the matrix of the printed formulation during disintegration testing. However, significantly lower disintegration times were observed for the *Ref* samples (p=0.034) compared to the *Amb* samples, attributed to the swollen matrix [55] and mobile polymer chains of the former [56]. Additionally, Zheng et al. [57] reported that forms stored in cooler conditions generally disintegrate faster than those stored in warmer conditions. Nevertheless, neither the storage condition nor the length of storage increased the disintegration time above 180 s, indicating that the forms remained within acceptable disintegration limits for this study.

Furthermore, due to the possible presence of the hydrated form of caffeine in the matrix, the loss of water and/or potential re-crystallization would affect the drug content and mass of the printed object [58,59,60]. Although, the effect of the length of storage on the latter was not significant (Figure A5b), it was however not the case for the former. Therefore, as shown in Figure 5c, there was a significant difference on the drug content of the printed forms over the length of storage (p=0.0128). The potential degradation of caffeine under the storage conditions may also be another explanation for this observation (discussed in Section 2.4). Interestingly, despite the statistical difference, the predicted drug content of the printed objects were within 90–110% of the label claim (3.4 mg) throughout the study.

### 2.4. Forced Degradation Studies

**Figure 6:**
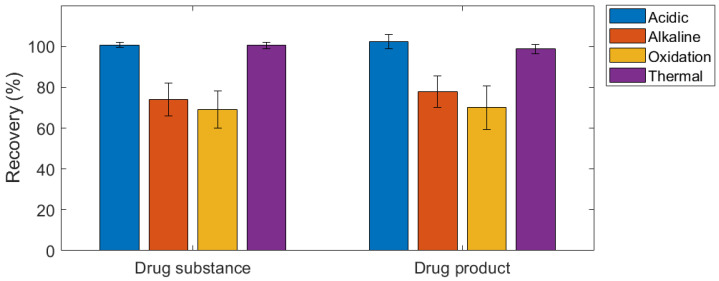
Percentage recovery of caffeine in drug substance and 3D printed dosage form.

Generally, the behaviour of caffeine under stress conditions was similar for both the drug substance and drug product (Figure 6). The API (Retention time, Rt = 9.54 min) in the drug substance solution, shown in Figure A6a, was stable under thermal stress conditions with neither a change in the peak area nor new peaks observed. The percentage recovery was approximately 100%. Similarly, the API in the drug product, shown in Figure A6b, was considerably stable on exposure to thermal stress with a percentage recovery of 99%. As shown in Figure 6 and Figure A7, there was minimal peak reduction of the API both in the drug substance solution and drug product under acidic stress conditions (Rt = 9.54 min). However, a second peak emerged in the drug product’s chromatogram (Rt = 5.84 min), suggesting potential instability of the excipients in acidic environments (Figure A7b). Regarding stability under alkali stress conditions, 26% and 22% of the drug degraded in the drug substance and drug product, respectively, with the generation of a new peak at Rt = 6.45 min for the API and Rt = 6.30 min for the drug product (Figure A8). Similarly, oxidative stress led to a reduction in the peak area of caffeine in both the drug substance and drug product (Figure A9). The percentage degradation observed was 31% and 30%, with the appearance of a new peak at Rt = 6.02 min for the API and Rt = 5.90 min for the drug product.

In comparison, the samples analysed during stability testing, as shown in Figure A10, exhibited none of the degradant peaks identified in the forced degradation studies. This absence suggests either insufficient degradation of the product during the evaluated storage conditions or that the precautions taken, such as storing the printed formulations in plastic containers and minimizing air exposure during handling, were effective in preventing potential chemical degradation from the conditions identified above. However, thermal stress appears to potentially influence the reduction of the API in the presence of excipients. In both the degradation and stability studies, a reduction in the caffeine peak was observed in the drug product, without the identification of a corresponding degradation peak.

**Figure 7:**
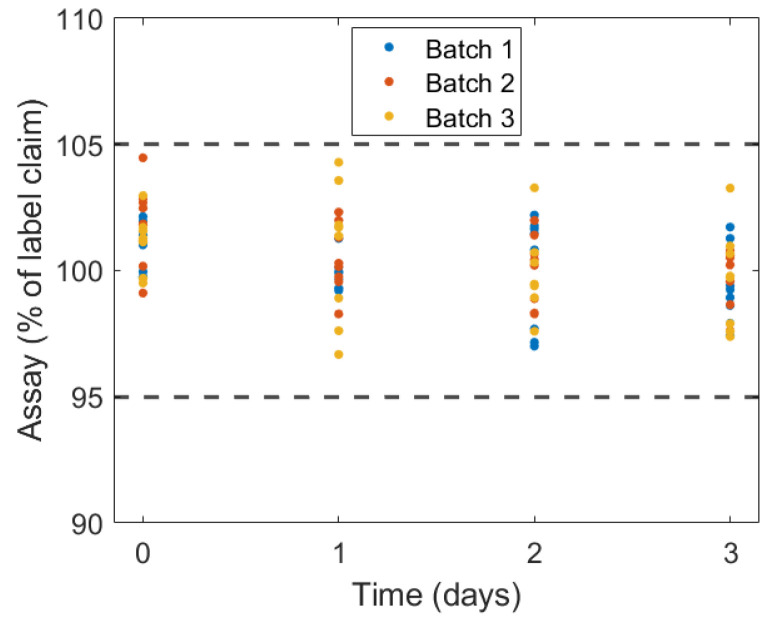
Variation of the drug recovery of the dosage form containing 3.4 mg across the length of storage.

However, due to the lack of control over the microbiological quality of the environment during the processes of printing, handling, and testing of the object, extended stability studies were impractical. In addition, the goal was to print gummy formulations; thus, drying to reduce moisture content and, consequently, microbial growth was not desired. The use of an antimicrobial agent could potentially extend the shelf-life of the drug product. Nevertheless, 3D-printed drugs are typically designed for on-demand printing and therefore may not require extended storage. This on-demand usage model thus nullifies any variability that different storage lengths and conditions may introduce. Additionally, given the broad therapeutic range of caffeine in preterm newborns [61,62,63], the observed variability in drug content across different time points may be considered acceptable. Moreover, as shown in Figure 7, the variation in the percentage label claim across and within the tested time points and batches remained within 5% of the label claim in all cases, underscoring the repeatability and precision of the 3D printing process itself.

### 2.5. ATR-FTIR

**Figure 8:**
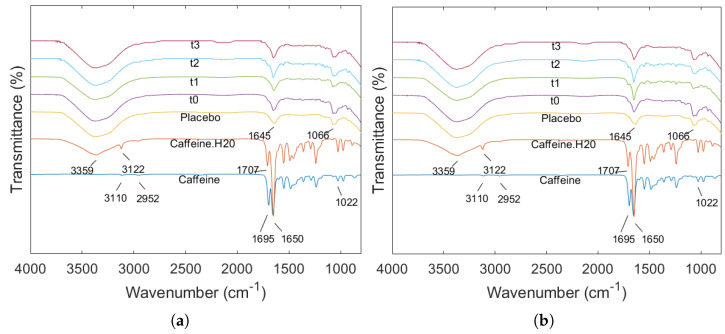
ATR-FTIR spectra of anhydrous caffeine, caffeine hydrate, drug loaded placebos and printed objects across the length of storage, for samples stored at 25 °C and 60% RH (**a**) and samples stored in a refrigerator (**b**). The time points investigated were immediately after printing (t0), after 1 (t1), 2 (t2) and 3 (t3) days.

Figure 8 presents the ATR-FTIR results of anhydrous caffeine, hydrated caffeine crystals, the drug-free placebo measured on day 1 of the stability study, and printed formulations measured at various time points. The FTIR spectrum of caffeine shows the aromatic C-H stretch at 3110 cm^−1^ and 2952 cm^−1^ [64,65,66]. The peaks at 1695 cm^−1^ and 1650 cm^−1^ are due to C=O and C=N stretching of cyclic hydrocarbons, respectively. The peak at 1022 cm^−1^ is likely due to the >C=O (ketonic) group stretching. In contrast, due to the presence of water in the hydrated caffeine crystals, shifts of these peaks were observed (from 3110 cm^−1^ and 1695 cm^−1^ to 3122 cm^−1^ and 1707 cm^−1^, respectively). In addition to these peaks, there was a peak at 3359 cm^−1^ due to the O-H stretch from water in the hydrated caffeine samples [65,67].

Similar peaks were observed in the drug-free placebo, samples stored at 25 °C and 60% RH (Figure 8a), and samples stored in the refrigerator (Figure 8b). Notably, the O-H stretch from water at 3359 cm^−1^ was present in the samples at the different time points as well as the placebo. However, there was the appearance of a peak at 1066 cm^−1^ in the printed formulations masking the peaks of the vibrations of the ketonic groups of caffeine at 1022 cm^−1^. This peak corresponds to the C-O stretching of the glycosidic bonds of the carbohydrate excipients (see Figure A11). Also, the absence of the peak corresponding to C=O stretching at 1695 or 1707 cm^−1^, suggests interactions between caffeine and the other components in the mixture. Specifically, the hydroxyl groups in agar, HPMC, and sucrose can form hydrogen bonds with the carbonyl groups of caffeine [68]. Additionally, the formation of complexes involving the carbonyl group in strong intermolecular interactions can change its vibrational characteristics, leading to the disappearance of the C=O stretching peak [69]. Both of these could potentially explain the reduction of the drug content across the length of the storage and why none of the degradation products from the degradation study were observed [70,71].

## 3. Conclusions

This study highlights the application of semi-solid extrusion 3D printing for designing personalisable gel-based dosage forms tailored for point-of-care settings. The gel-based dosage forms consisting of agar and hydroxypropyl methylcellulose (HPMC) exhibited immediate release of caffeine and met the quality standards expected of conventionally manufactured products. Regression analysis of theoretical volume and drug content helped estimate the dimensions required to produce a 3.4 mg dose of caffeine suitable for an infant weighing 1.36 kg. The linear regression equation estimated that a cylindrical object with a diameter of 4 mm and a height of 2.8 mm would contain the desired drug content. This prediction was accurate and the percentage of caffeine recovered from the printed object was 103.46%. However, within a 95% confidence interval, the estimated drug content of such forms could vary from 2.84 mg to 5.16 mg, indicating potential variability that might be minimised with more extensive data fitting. Despite this, the differences observed among three batches of printed objects with these dimensions were statistically insignificant. In contrast, significant discrepancies were noted between the theoretical and actual volumes of the printed objects, and both the volume and disintegration time varied markedly under the two storage conditions assessed: refrigerated and at 25 °C and 60% relative humidity. Additionally, drug content and disintegration time of the dosage forms exhibited substantial changes over a three-day storage period. Despite these variations, forced degradation studies revealed no degradation peaks in the stored samples, suggesting minimal chemical degradation of caffeine in the gel-matrix under the tested conditions. However, attenuated Fourier transform infrared spectroscopy indicated possible interactions between the hydrated form of caffeine and the excipients, which might account for the observed reduction in drug content over time. Overall, this study successfully demonstrates the potential of 3D printing technology for producing precise and accurate doses of caffeine, particularly for on-demand applications in clinical settings. Despite some challenges related to storage stability and variability, the results highlight the potential of 3D-printed agar and HPMC dosage forms for pharmaceutical applications.

## 4. Materials & Methods

### 4.1. Materials

Agar, anhydrous caffeine and pulverised sucrose were purchased from Cooper’s Laboratory, France and Methocel ^®^ K4M (HPMC, USP 2208 grade, 4000 cp, Controlled release (CR) Premium, MW = $2.1\times 10^{5}$ g/mol, 8.1% hydroxypropyl and 22% methoxy substituted) was gifted by Colorcon Ltd, France. Ultrapure water for sample preparation was obtained from a Purelab^®^ water purification system (VWR, Rosny-sous-Bois, France) at resistance of 18 MΩ×cm. Hydrochloric acid (HCl), hydrogen peroxide (H_2_O_2_) and sodium hydroxide (NaOH) were purchased from VWR, France. High-performance liquid chromatography (HPLC) grade methanol and acetonitrile were procured from Merck, France. All the solutions were filtered through a 0.45 µm membrane (Merck, Lyon, France) before their use.

### 4.2. Preparation of Hydrogels

As reported in our previous study [72], hydrogels were prepared by mixing 3 g of agar, 2 g of HPMC, 10 g of sucrose (added to improve palatibility), 2 g of caffeine. A drug free placebo was also prepared without caffeine. The weighed powders were homogenised using a laboratory spatula and while stirring, ultrapure water (heated to 98 °C) was added to the powder mixture, to a total of 100 g. The beaker containing the formulation was then placed in a water bath at 98 °C until an amber-coloured highly viscous solution was formed. After which the beaker was taken out and left at room temperature (T = 20 °C) until the gel was formed. While heating at 98 °C would not be suitable for thermosensitive APIs, alternative methods, such as swelling the gel in a solution containing these sensitive ingredients, can be employed to prevent heat exposure [73,74]. However, in this study, such elevated temperature was essential for ensuring that caffeine was adequately dissolved within the gel matrix.

### 4.3. Printing Process

One of the objectives of this study was to ensure the printing of precise individualised dosages for paediatric patients, a calibration of the printing process was thus performed by printing cylindrical objects with different dimensions, as shown in Table 1. The dimensions were chosen arbitrarily while keeping a diameter to height aspect ratio from 1.5 to 2. The printing was performed using a Delta WASP 2040 printer equipped with a clay extruder at room temperature. The layer height was set to 0.4 mm, the infill pattern was rectilinear, and the infill density was 100%. The nozzle diameter used in this study was 0.84 mm and the print head speed was 20 mm/s. The obtained tablets were used without any additional drying, and their weights were recorded immediately after printing using an analytical balance (Sartorius^™^ BCE224I-1S, Aubagne, France). The obtained drug content was then plotted against the theoretical volume and a linear regression was performed based on the data points. The resulting regression equation was then used to predict the dimensions of a dosing unit that contains 3.4 mg of caffeine based on a dose of 2.5 mg/kg for the birth weight of a preterm infant weighing 1.36 kg. The selection of this weight was based on the study of Ye et al. [75], where an increased risk of adverse reactions was associated with birth weight and serum concentration of theophylline.

### 4.4. Method of Assay

Before the assay, the gel-based dosage form was liquefied by dissolving it in 50 mL of purified water. The resulting solution was then transferred to 50 mL amber glass bottles to protect it from light exposure. To ensure complete dissolution and uniformity of the sample, the sealed bottles were heated to a temperature of 60 °C and were left at this temperature for 1 h. The solution containing the dissolved gel was then centrifuged at 4000 rpm (SIGMA 2-16P, Osterode am Harz, Germany) for 5 min to ensure the sedimentation of any floating particles.

**Figure A1:**
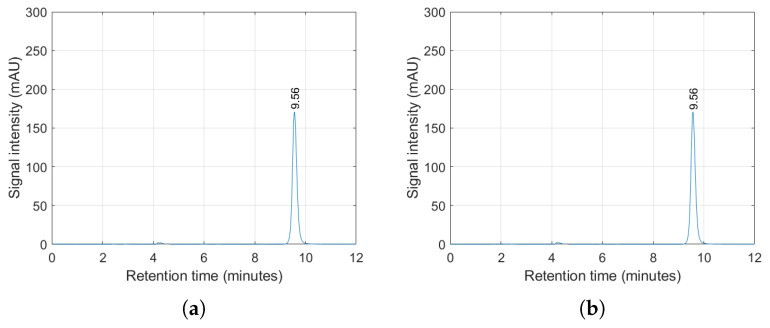
Chromatogram of caffeine (**a**) and caffeine-loaded gel formulation dissolved in water (**b**).

**Figure A2:**
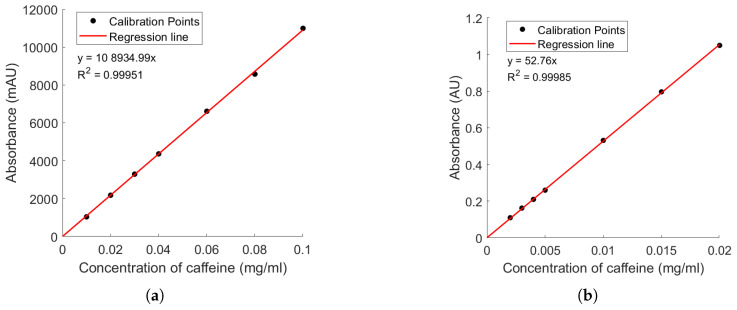
Calibration curve, equation and coefficient of correlation, $R^{2}$ of HPLC (**a**) and UV assay (**b**).

The HPLC assay was developed using an Agilent technologies HPLC system (1260 Infinity II series, France) equipped with a Quaternary Pump (G7111B), Auto Vial Sampler (G7129A), Column Chamber (G7116A), Diode Array Detector DAD WR (G7115A). The column’s oven was maintained at 30 °C. Isocratic elution was performed using methanol and water (40:60, *v*/*v*). The overall run time was 12 min, and the flow rate was 0.5 mL/min. 20 μL of the supernatant was filtered and injected into the HPLC system, which was set at a detection wavelength of 272 nm. The specificity at this wavelength was evaluated by injecting similar concentrations of caffeine in water and caffeine contained in gel formulation. The retention peaks and time in both cases were similar (Figure A1), indicating that none of the excipients influenced the absorption of caffeine at the selected wavelength. For the ultraviolet (UV) assay, the diluted supernatant was analysed using an Agilent UV spectrophotometer (Cary 8454, France). The sample was placed in a quartz cuvette with a path length of 1 cm and the absorbance was also set at 272 nm. Using the developed HPLC method, a calibration curve ranging from 0.01 to 0.1 mg/mL ($R^{2}$ = 0.9999) was developed by diluting 1 mg/mL solution of caffeine with the solvent, ultra-purified water (Figure A2a).

On the other hand, the UV assay was used to construct a calibration curve ranging from 0.002 to 0.02 mg/mL ($R^{2}$ = 0.9999) was developed by diluting 1 mg/mL solution of caffeine with the solvent, ultra-purified water (Figure A2b). The HPLC assay was used for the stability study to ensure the identification of potential degradation. The UV assay, on the other hand, was used for the analysis of the drug content and the dissolution study.

### 4.5. Evaluation of Quality Control Attributes

#### 4.5.1. Calculation of Volumes and Dimensional Measurement of Printed Objects

The theoretical volumes of the models (volume of a cylindrical-shaped object) were calculated using the dimensions of the 3D models shown in Table 1. The size of the forms after printing (n = 10) was measured using a digital vernier calliper (Micron, France) and the relative standard deviation (RSD) of the samples as well as their volumes were calculated (volume of a cylindrical-shaped object).

#### 4.5.2. Uniformity of Mass

The accuracy of the printing process was evaluated using the mass variation of printed forms according to the Ph. Eur. 2.9.5 20905 (04/2023). Printed forms (n = 10) were weighed individually, and the arithmetic mean, and RSD were calculated.

#### 4.5.3. Uniformity of Dosage Units

The uniformity of dosage units (n = 10) was evaluated according to the Ph. Eur. 2.9.40 20940 (04/2017). According to the monograph, the acceptance value (AV) was calculated for each batch and should not exceed 15.

#### 4.5.4. Disintegration Time

The disintegration time of printed forms was analysed according to the Ph. Eur. 2.9.1, 20901 (01/2022). A manual tablet disintegration tester (DT50, Sotax AG, Aesch, Switzerland) was used. Printed forms (n = 6) per batch were analysed and the individual disintegration times and the standard deviation (SD) was calculated.

#### 4.5.5. Dissolution Study

The in vitro dissolution study was conducted using an Xtend^™^ apparatus (Sotax, Aesch, Switzerland). For each dose, printed forms (n = 6) were placed into separate baskets. These baskets were placed in dissolution flasks containing 900 mL of ultra-purified water at 37 °C and constantly stirred at 100 rpm. 3 mL samples were withdrawn through a 0.45 µm membrane filter at predetermined times and the calculation was corrected for the withdrawn solvent.

### 4.6. Identification of Variability

To ensure precise and accurate on-demand printing, the variabilities that may occur as a result of the batch of feedstock, the length of storage and the storage condition were investigated. The two storage conditions investigated were: 25 ± 2 °C at 60% ± 5% relative humidity, RH (Amb) and at a refrigerator at 5 ± 3 °C (Ref). The three batches (B1, B2 and B3) at four time points were investigated. The time points investigated were immediately after printing (t0), after 1 (t1), 2 (t2) and 3 (t3) days.

The rheological characterization of the feedstock was performed using a ThermoHaake RheoStress 600 (RS600) rheometer with plate–plate geometry (diameter = 35 mm) at a gap height of 0.5 mm, set based on the sample and test. The viscoelastic behaviour of the samples was evaluated through oscillatory tests, recording only the phase angle (δ in °) at the linear viscoelastic region. δ, which represents the balance between solid and liquid properties, was the most relevant parameter for evaluating printability based on a previous study and was therefore used to evaluate viscoelasticity [72]. All measurements were performed in duplicates at 20 °C.

**Figure A3:**
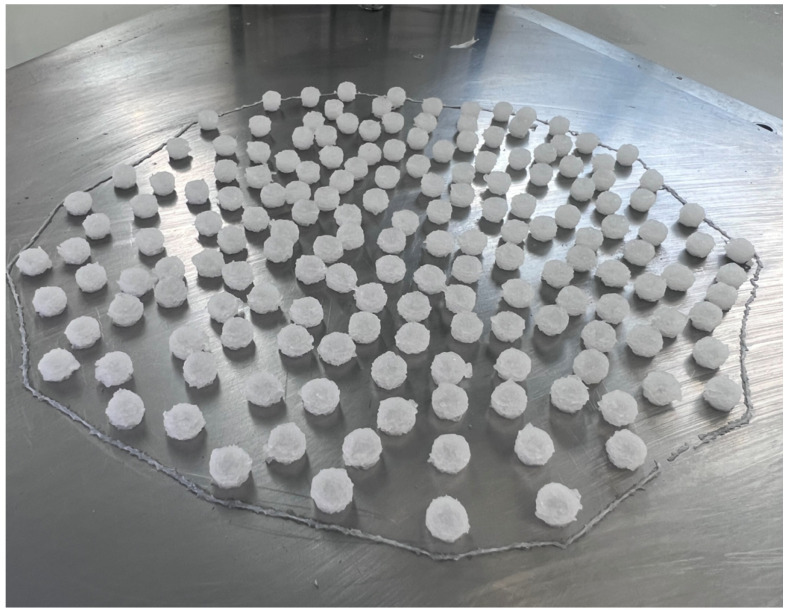
Printed objects containing 3.4 mg of caffeine.

**Figure A4:**
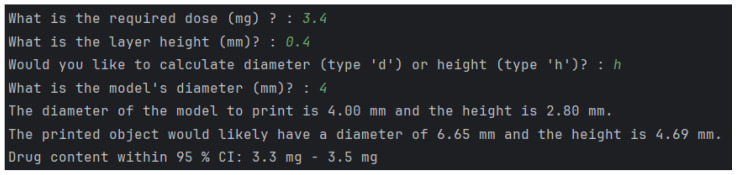
Calculation of model and printed size of a cylindrical shaped dosage unit containing 3.5 mg of caffeine.

**Figure A5:**
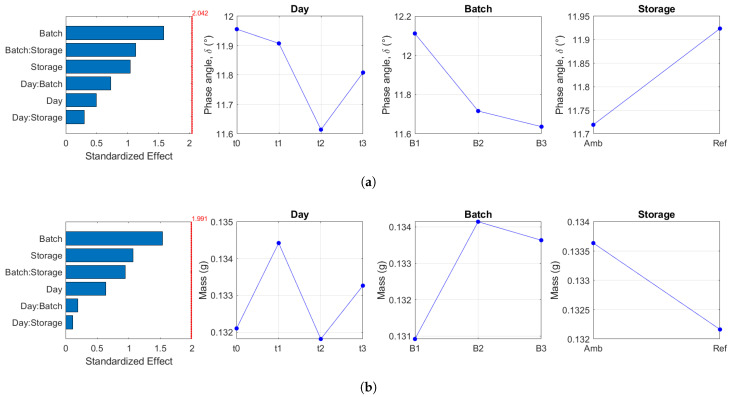
Standardised effects of the factors on the phase angle of the feedstock (**a**) and the mass of the printed objects (**b**). The time points investigated were immediately after printing (t0), after 1 (t1), 2 (t2) and 3 (t3) days. The dotted red line corresponds to the critical value; effects exceeding this threshold are considered statistically significant.

**Figure A6:**
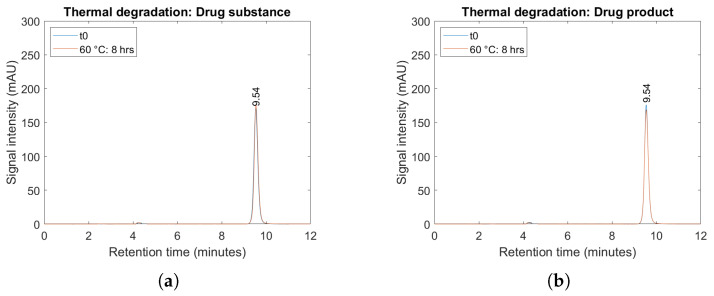
Forced degradation of API (**a**) and gummy formulation (**b**) at 60 °C for 8 h. Caffeine’s peak was observed at Rt of 9.54 min, no additional peak was observed.

**Figure A7:**
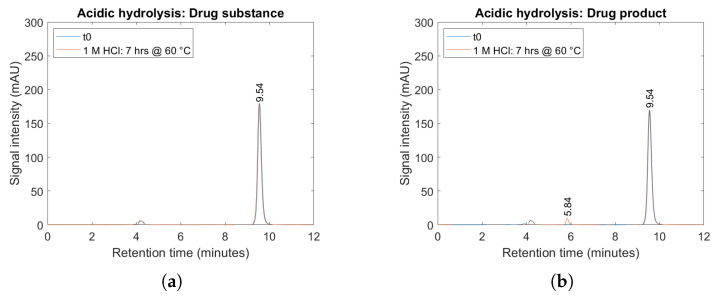
Forced degradation of API (**a**) and gummy formulation (**b**) in 1 M HCl at 60 °C for 7 h. Caffeine’s peak was observed at Rt of 9.54 min for both API and gummy formulation, and degradant’s peak was observed at Rt 5.84 min for gummy formulation.

**Figure A8:**
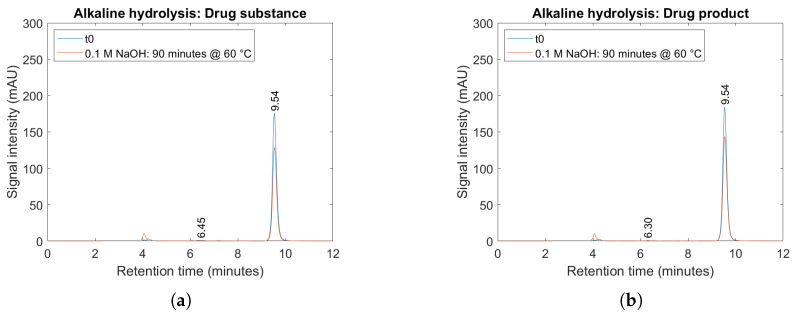
Forced degradation of API (**a**) and gummy formulation (**b**) in 0.1M NaOH at 60 °C for 90 min. Caffeine’s peak was observed at Rt of 9.54 min and the degradant’s peak was observed at Rt 6.45 and 6.30 min for API and gummy formulation, respectively.

**Figure A9:**
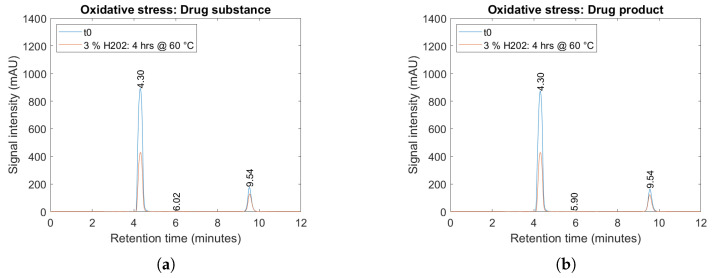
Forced degradation of API (**a**) and gummy formulation (**b**) in 3% H_2_O_2_ at 60 °C for 4 h. Caffeine’s peak was observed at Rt of 9.54 min and the degradant’s peak was observed at Rt 6.02 and 5.90 min for API and gummy formulation, respectively.

**Figure A10:**
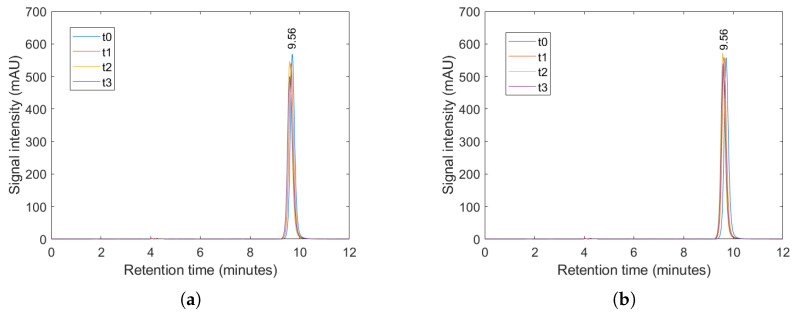
Chromatograms of samples evaluated during the stability study. Shown here is the third batch (n = 4), for samples stored at 25 °C at 60% (**a**) and samples stored in the refrigerator (**b**). Caffeine’s peak was observed at Rt of 9.54 min, no additional peak was observed.

**Figure A11:**
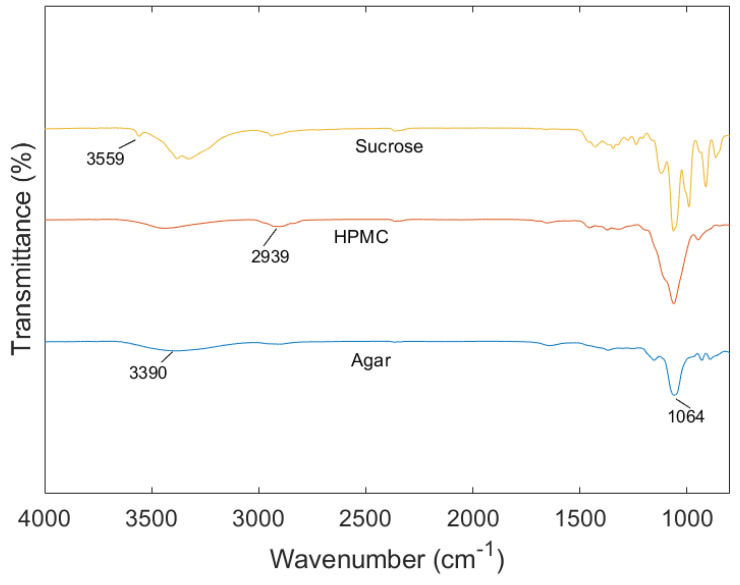
ATR-FTIR spectra of agar, HPMC and sucrose.

For the dosage form, objects (n = 120) containing 3.4 mg of caffeine were printed in the form of cylinders with a 4 mm diameter and 2.8 mm height (Figure A3). API content was measured immediately using HPLC after printing which was considered as the baseline (t0). The acceptance criterion for the stability was that all samples had to be between 95–105% of the desired drug content (3.4 mg). The samples were also examined for possible physical alterations by direct sample observation on a white background. Other similar quality control attributes as detailed in Section 4.5 were also performed at each time point for at least three samples.

### 4.7. Forced Degradation Study

Previous studies mention the potential degradation of caffeine under acidic, alkaline, oxidative and wet heat conditions [76,77]. Based on these studies, forced degradation studies were performed under acidic, alkaline, oxidative and wet heat conditions to detect potential degradation. For this, a stock solution of caffeine (drug substance) was diluted to 60 µg/mL and the 3D printed formulation (drug product) containing 60 µg/mL of caffeine was assessed under acidic, alkaline, oxidative and wet heat conditions. The solutions of the drug substance and drug product were mixed with each degradation medium (water, 1 M HCl, 0.1 M NaOH, 3% H_2_O_2_) in a 1:1 ratio (*v*/*v*). All stressed samples were cooled, neutralised (when applicable), and diluted to 20 µg/mL using ultrapure water (diluting solvent). The test was conducted at least 3 times using 3 different sample preparations and analysed using the HPLC method described in Section 4.4. The stressed sample solutions were compared against the freshly prepared solutions, and the recovery of the drug presented.

### 4.8. ATR-FTIR

Infrared spectra of anhydrous caffeine, caffeine monohydrate formed by the cooling of hot supersaturated caffeine solution and the 3D printed forms over the length of storage and at the different storage conditions were obtained using an ATR-FTIR (Agilent Cary 630 FTIR) spectrometer. The scanning range was 4000–400 cm^−1^ at the resolution of 4 cm^−1^ and the spectrum obtained was treated with the program function of baseline correction. Each sample was measured in duplicate with a third measurement performed in case of inconsistencies. The measurement was performed across the 3 batches and the averages of the measurements are presented.

### 4.9. Data Analysis

Data analysis was conducted using MATLAB R2023a (MathWorks, Natick, MA, USA). The variability of three batches (batch) of feedstock and printed objects containing 3.4 mg of caffeine was evaluated using analysis of variance (ANOVA) across four time points (day), two different storage conditions (storage), and their interactions (day:batch, day:storage and batch:storage). Significant difference was set at α = 5% (*p*-value, *p* < 0.05). In addition, the similarity factor, $f_{2}$ between the dissolution value of the reference ($R_{t}$) and test product ($T_{t}$) at time t based on the number of time points, n was calculated using Equation (1) [78]. An $f_{2}$ value that is greater than 50 is generally considered acceptable.

(1) $$f_{2}=50\times \log\left[{\left(1+\frac{1}{n}\sum_{t=1}^{n}{\left(R_{t}-T_{t}\right)}^{2}\right)}^{-0.5}\times 100\right]$$

## Figures and Tables

**Table 1:** Dimensions of 3D models and printed objects.

Notation	Model		Printed Object			
			Average		RSD	
	Diameter (mm)	Height (mm)	Diameter (mm)	Height (mm)	Diameter (%)	Height (%)
C1	4	2.4	5.9	4.0	1.1	3.0
C2	5	2.4	8.5	4.7	2.5	4.1
C3	6.9	3.2	11.0	5.8	1.9	1.7
C4	7	3.6	11.5	6.0	1.5	3.2
C5	8	5.2	14.1	8.2	2.4	4.0

**Table 2:** Attributes of printed gel-based dosage forms.

Notation		Mass		Disintegration Time		Drug Content	
	SA/V (mm^−1^)	Average (g)	RSD (%)	Average (s)	SD (s)	Label Claim (mg of Caffeine)	Acceptance Value (AV)
C1	1.18	0.125	2.4	65	11	2.8	0.1
C2	0.89	0.241	2.5	67	4	4.7	0.3
C3	0.71	0.563	1.9	82	3	11.2	0.7
C4	0.68	0.704	2.2	83	6	13.6	0.6
C5	0.53	1.219	2.0	345	9	23.8	1.3

## Data Availability

The raw data used for the analysis of variance (ANOVA) in this study is available here: Recherche Data Gouv (accessed on 30 September 2024).

## Appendix A

The following are the Supplementary data to this article:

## References

[1] Willson C. (2018). The Clinical Toxicology of Caffeine: A Review and Case Study. Toxicol. Rep..

[2] Nonappa N., Kolehmainen E. (2016). Caffeine as a Gelator. Gels.

[3] Bright M., Raman V., Laupland K.B. (2021). Use of Therapeutic Caffeine in Acute Care Postoperative and Critical Care Settings: A Scoping Review. BMC Anesthesiol..

[4] Dobson N.R., Hunt C.E. (2013). Pharmacology Review: Caffeine Use in Neonates: Indications, Pharmacokinetics, Clinical Effects, Outcomes. NeoReviews.

[5] Abdel-Hady H., Nasef N., Shabaan A.E., Nour I. (2015). Caffeine Therapy in Preterm Infants. World J. Clin. Pediatr..

[6] Shrestha B., Jawa G. (2017). Caffeine Citrate—Is It a Silver Bullet in Neonatology?. Pediatr. Neonatol..

[7] McCloskey A.P., Bracken L., Vasey N., Ehtezazi T. (2023). 3D Printing—An Alternative Strategy for Pediatric Medicines. Expert Rev. Clin. Pharmacol..

[8] Ullah M., Wahab A., Khan S.U., Naeem M., ur Rehman K., Ali H., Ullah A., Khan A., Khan N.R., Rizg W.Y. (2023). 3D Printing Technology: A New Approach for the Fabrication of Personalized and Customized Pharmaceuticals. Eur. Polym. J..

[9] Lafeber I., Ruijgrok E.J., Guchelaar H.J., Schimmel K.J.M. (2022). 3D Printing of Pediatric Medication: The End of Bad Tasting Oral Liquids?—A Scoping Review. Pharmaceutics.

[10] Xue A., Li W., Tian W., Zheng M., Shen L., Hong Y. (2023). A Bibliometric Analysis of 3D Printing in Personalized Medicine Research from 2012 to 2022. Pharmaceuticals.

[11] Funk N.L., Leão J., de Oliveira T.V., Beck R.C.R., Banerjee S. (2023). Semi-Solid Extrusion (SSE) in Pharmaceuticals. Additive Manufacturing in Pharmaceuticals.

[12] Zhang B., Belton P., Yi Teoh X., Gleadall A., Bibb R., Qi S. (2024). An Investigation into the Effects of Ink Formulations of Semi-Solid Extrusion 3D Printing on the Performance of Printed Solid Dosage Forms. J. Mater. Chem. B.

[13] Wang X.m., Li B., Zhang T., Li X.y. (2015). Performance of Nanofiltration Membrane in Rejecting Trace Organic Compounds: Experiment and Model Prediction. Desalination.

[14] Kulkarni U.D., Mahalingam R., Li X., Pather I., Jasti B. (2011). Effect of Experimental Temperature on the Permeation of Model Diffusants Across Porcine Buccal Mucosa. AAPS PharmSciTech.

[15] Johannesson J., Wu M., Johansson M., Bergström C.A.S. (2023). Quality Attributes for Printable Emulsion Gels and 3D-printed Tablets: Towards Production of Personalized Dosage Forms. Int. J. Pharm..

[16] Sjöholm E., Mathiyalagan R., Lindfors L., Wang X., Ojala S., Sandler N. (2022). Semi-Solid Extrusion 3D Printing of Tailored ChewTs for Veterinary Use—A Focus on Spectrophotometric Quantification of Gabapentin. Eur. J. Pharm. Sci..

[17] Zheng Z., Lv J., Yang W., Pi X., Lin W., Lin Z., Zhang W., Pang J., Zeng Y., Lv Z. (2020). Preparation and Application of Subdivided Tablets Using 3D Printing for Precise Hospital Dispensing. Eur. J. Pharm. Sci..

[18] Roche A., Sanchez-Ballester N.M., Aubert A., Rossi J.C., Begu S., Soulairol I. (2023). Preliminary Study on the Development of Caffeine Oral Solid Form 3D Printed by Semi-Solid Extrusion for Application in Neonates. AAPS PharmSciTech.

[19] O’Brien F., Clapham D., Krysiak K., Batchelor H., Field P., Caivano G., Pertile M., Nunn A., Tuleu C. (2019). Making Medicines Baby Size: The Challenges in Bridging the Formulation Gap in Neonatal Medicine. Int. J. Mol. Sci..

[20] Krueger L., Cao Y., Zheng Z., Ward J., Miles J.A., Popat A. (2023). 3D Printing Tablets for High-Precision Dose Titration of Caffeine. Int. J. Pharm..

[21] Liu B., Chen K. (2024). Advances in Hydrogel-Based Drug Delivery Systems. Gels.

[22] Ribeiro M., Simões M., Vitorino C., Mascarenhas-Melo F. (2024). Hydrogels in Cutaneous Wound Healing: Insights into Characterization, Properties, Formulation and Therapeutic Potential. Gels.

[23] Kaliampakou C., Lagopati N., Pavlatou E.A., Charitidis C.A. (2023). Alginate–Gelatin Hydrogel Scaffolds; An Optimization of Post-Printing Treatment for Enhanced Degradation and Swelling Behavior. Gels.

[24] Dabbaghi A., Ramazani A., Farshchi N., Rezaei A., Bodaghi A., Rezayati S. (2021). Synthesis, Physical and Mechanical Properties of Amphiphilic Hydrogels Based on Polycaprolactone and Polyethylene Glycol for Bioapplications: A Review. J. Ind. Eng. Chem..

[25] Saqib M.N., Khaled B.M., Liu F., Zhong F. (2022). Hydrogel Beads for Designing Future Foods: Structures, Mechanisms, Applications, and Challenges. Food Hydrocoll. Health.

[26] Wang J., Liu Y., Zhang X., Rahman S.E., Su S., Wei J., Ning F., Hu Z., Martínez-Zaguilán R., Sennoune S.R. (2021). 3D Printed Agar/ Calcium Alginate Hydrogels with High Shape Fidelity and Tailorable Mechanical Properties. Polymer.

[27] Ramli N.A., Adam F., Mohd Amin K.N., Nor A.M., Ries M.E. (2023). Evaluation of Mechanical and Thermal Properties of Carrageenan/Hydroxypropyl Methyl Cellulose Hard Capsule. Can. J. Chem. Eng..

[28] Kittipongpatana O.S., Trisopon K., Wattanaarsakit P., Kittipongpatana N. (2022). Fabrication and Characterization of Orodispersible Composite Film from Hydroxypropylmethyl Cellulose-Crosslinked Carboxymethyl Rice Starch. Membranes.

[29] Cavelier M., Gondé H., Costa D., Lamoureux F., Pereira T., Buchbinder N., Varin R., Hervouët C. (2023). Development of an Oral Liquid Formulation of Nicardipine Hydrochloride Compounded with Simple Excipients for the Treatment of Pediatric Hypertension. Pharmaceutics.

[30] Su H., Yang S., Chen S., Chen X., Guo M., Zhu L., Xu W., Liu H. (2024). What Happens in the Gut during the Formation of Neonatal Jaundice—Underhand Manipulation of Gut Microbiota?. Int. J. Mol. Sci..

[31] Radwan I.M., Sakr M.M.A., Mohamed S.A. (2023). Is Oral Agar Combined with Phototherapy Superior than Phototherapy in Treatment of Neonatal Indirect Hyperbilirubinemia. Sci. J. Med Sch..

[32] Blessy M., Patel R.D., Prajapati P.N., Agrawal Y.K. (2014). Development of Forced Degradation and Stability Indicating Studies of Drugs—A Review. J. Pharm. Anal..

[33] Shah U., Kavad M., Raval M. (2014). Development and Validation of Stability-indicating RP-HPLC Method for Estimation of Pamabrom in Tablets. Indian J. Pharm. Sci..

[34] Sonawane S., Jadhav S., Rahade P., Chhajed S., Kshirsagar S. (2016). Development and Validation of Stability-Indicating Method for Estimation of Chlorthalidone in Bulk and Tablets with the Use of Experimental Design in Forced Degradation Experiments. Scientifica.

[35] Baertschi S.W., Huynh-Ba K. (2010). Forced Degradation and Its Relation to Real Time Drug Product Stability. Pharmaceutical Stability Testing to Support Global Markets.

[36] Bom S., Ribeiro R., Ribeiro H.M., Santos C., Marto J. (2022). On the Progress of Hydrogel-Based 3D Printing: Correlating Rheological Properties with Printing Behaviour. Int. J. Pharm..

[37] Sastri T.K., Gupta V.N., Chakraborty S., Madhusudhan S., Kumar H., Chand P., Jain V., Veeranna B., Gowda D.V. (2022). Novel Gels: An Emerging Approach for Delivering of Therapeutic Molecules and Recent Trends. Gels.

[38] Kaur M., Sharma A., Puri V., Aggarwal G., Maman P., Huanbutta K., Nagpal M., Sangnim T. (2023). Chitosan-Based Polymer Blends for Drug Delivery Systems. Polymers.

[39] Jia D., Muthukumar M. (2021). Theory of Charged Gels: Swelling, Elasticity, and Dynamics. Gels.

[40] Es Sayed J., Khoonkari M., Oggioni M., Perrin P., Sanson N., Kamperman M., Włodarczyk-Biegun M.K. (2022). Multi-Responsive Jammed Micro-Gels Ink: Toward Control over the Resolution and the Stability of 3D Printed Scaffolds. Adv. Funct. Mater..

[41] Laffleur F., Keckeis V. (2020). Advances in Drug Delivery Systems: Work in Progress Still Needed?. Int. J. Pharm..

[42] Goyanes A., Robles Martinez P., Buanz A., Basit A.W., Gaisford S. (2015). Effect of Geometry on Drug Release from 3D Printed Tablets. Int. J. Pharm..

[43] Khizer Z., Akram M.R., Sarfraz R.M., Nirwan J.S., Farhaj S., Yousaf M., Hussain T., Lou S., Timmins P., Conway B.R. (2019). Plasticiser-Free 3D Printed Hydrophilic Matrices: Quantitative 3D Surface Texture, Mechanical, Swelling, Erosion, Drug Release and Pharmacokinetic Studies. Polymers.

[44] Domsta V., Hänsch C., Lenz S., Gao Z., Matin-Mann F., Scheper V., Lenarz T., Seidlitz A. (2023). The Influence of Shape Parameters on Unidirectional Drug Release from 3D Printed Implants and Prediction of Release from Implants with Individualized Shapes. Pharmaceutics.

[45] Funk N.L., Fantaus S., Beck R.C.R. (2022). Immediate Release 3D Printed Oral Dosage Forms: How Different Polymers Have Been Explored to Reach Suitable Drug Release Behaviour. Int. J. Pharm..

[46] Fanous M., Gold S., Muller S., Hirsch S., Ogorka J., Imanidis G. (2020). Simplification of Fused Deposition Modeling 3D-printing Paradigm: Feasibility of 1-Step Direct Powder Printing for Immediate Release Dosage Form Production. Int. J. Pharm..

[47] Onsawai P., Phetpan K., Khurnpoon L., Sirisomboon P. (2021). Evaluation of Physiological Properties and Texture Traits of Durian Pulp Using Near-Infrared Spectra of the Pulp and Intact Fruit. Measurement.

[48] Malik A.N., Khan S.A., Lazoglu I. (2020). A Novel Hybrid Frost Detection and Defrosting System for Domestic Refrigerators. Int. J. Refrig..

[49] Metze F.K., Sant S., Meng Z., Klok H.A., Kaur K. (2023). Swelling-Activated, Soft Mechanochemistry in Polymer Materials. Langmuir.

[50] Krakovský I., Hanyková L., Štastná J. (2024). Phase Transition in Polymer Hydrogels Investigated by Swelling, DSC, FTIR and NMR. J. Therm. Anal. Calorim..

[51] Tillinghast G., Sánchez-Rivera K.L., Huber G.W., Winter H.H., Rothstein J.P. (2024). Shear and Extensional Rheology of Polyethylenes Recycled Using a Solvent Dissolution Process. Rheol. Acta.

[52] Li S., Zhang M., Vogt B.D. (2022). Delayed Swelling and Dissolution of Hydrophobically Associated Hydrogel Coatings by Dilute Aqueous Surfactants. ACS Appl. Polym. Mater..

[53] Nokhodchi A., Javadzadeh Y. (2007). The Effect of Storage Conditions on the Physical Stability of Tablets. Pharm. Technol. Eur..

[54] Markl D., Maclean N., Mann J., Williams H., Abbott A., Mead H., Khadra I. (2021). Tablet Disintegration Performance: Effect of Compression Pressure and Storage Conditions on Surface Liquid Absorption and Swelling Kinetics. Int. J. Pharm..

[55] Lenz J., Finke J.H., Bunjes H., Kwade A., Juhnke M. (2021). Tablet Formulation Development Focusing on the Functional Behaviour of Water Uptake and Swelling. Int. J. Pharm. X.

[56] Azevedo J.V.C., Hausnerova B., Möginger B., Sopik T. (2023). Effect of Chain Extending Cross-Linkers on the Disintegration Behavior of Composted PBAT/PLA Blown Films. Int. J. Mol. Sci..

[57] Zheng A.Y., Heng P.W.S., Chan L.W. (2022). Tablet Disintegratability: Sensitivity of Superdisintegrants to Temperature and Compaction Pressure. Pharmaceutics.

[58] Wang J., Li F., Lakerveld R. (2018). Process Intensification for Pharmaceutical Crystallization. Chem. Eng. Process. Process. Intensif..

[59] Andreeta M.R.B. (2012). Crystallization: Science and Technology.

[60] Javadzadeh Y., Hamedeyazdan S., Asnaashari S. (2012). Recrystallization of Drugs: Significance on Pharmaceutical Processing. Recrystallization.

[61] Aranda J.V., Beharry K.D. (2020). Pharmacokinetics, Pharmacodynamics and Metabolism of Caffeine in Newborns. Semin. Fetal Neonatal Med..

[62] Sugino M., Kuboi T., Noguchi Y., Nishioka K., Tadatomo Y., Kawaguchi N., Sadamura T., Nakano A., Konishi Y., Koyano K. (2023). Serum Caffeine Concentrations in Preterm Infants: A Retrospective Study. Sci. Rep..

[63] Long J.Y., Guo H.L., He X., Hu Y.H., Xia Y., Cheng R., Ding X.S., Chen F., Xu J. (2021). Caffeine for the Pharmacological Treatment of Apnea of Prematurity in the NICU: Dose Selection Conundrum, Therapeutic Drug Monitoring and Genetic Factors. Front. Pharmacol..

[64] Gunasekaran S., Sankari G., Ponnusamy S. (2005). Vibrational Spectral Investigation on Xanthine and Its Derivatives—Theophylline, Caffeine and Theobromine. Spectrochim. Acta Part Mol. Biomol. Spectrosc..

[65] González-González J., Zuñiga O., Hernández-Galindo M. (2017). Hydrated Solid Forms of Theophylline and Caffeine Obtained by Mechanochemistry. IOSR J. Pharm..

[66] Švecová M., Palounek D., Volochanskyi O., Prokopec V. (2020). Vibrational Spectroscopic Study of Selected Alkaloids with Therapeutic Effects. Spectrochim. Acta Part Mol. Biomol. Spectrosc..

[67] Paradkar M., Irudayaraj J. (2002). A Rapid FTIR Spectroscopic Method for Estimation of Caffeine in Soft Drinks and Total Methylxanthines in Tea and Coffee. J. Food Sci..

[68] Kwaśniewska-Sip P., Woźniak M., Jankowski W., Ratajczak I., Cofta G. (2021). Chemical Changes of Wood Treated with Caffeine. Materials.

[69] Świderski G., Kalinowska M., Gołębiewska E., Świsłocka R., Lewandowski W., Kowalczyk N., Naumowicz M., Cudowski A., Pietryczuk A., Nalewajko-Sieliwoniuk E. (2023). Structures, Antioxidant Properties, and Antimicrobial Properties of Eu(III), Gd(III), and Dy(III) Caffeinates and p-Coumarates. Molecules.

[70] Wang N., Sun H., Dong J., Ouyang D. (2021). PharmDE: A New Expert System for Drug-Excipient Compatibility Evaluation. Int. J. Pharm..

[71] Omari D.M., Akkam Y., Sallam A. (2021). Drug-Excipient Interactions: An Overview on Mechanisms and Effects on Drug Stability and Bioavailability. Ann. Rom. Soc. Cell Biol..

[72] Aina M., Baillon F., Sescousse R., Sanchez-ballester N.M., Begu S., Soulairol I., Sauceau M. (2024). Evaluation of the Printability of Agar and Hydroxypropyl Methylcellulose Gels as Gummy Formulations: Insights from Rheological Properties. Int. J. Pharm..

[73] Chew S.W.T., Shah A.H., Zheng M., Chang H., Wiraja C., Steele T.W.J., Xu C. (2020). A Self-Adhesive Microneedle Patch with Drug Loading Capability through Swelling Effect. Bioeng. Transl. Med..

[74] Zahra Q., Minhas M.U., Khan S., Wu P.C., Suhail M., Iqbal R., Bashir M. (2022). Fabrication of Polyethylene Glycol Hydrogels with Enhanced Swelling; Loading Capacity and Release Kinetics. Polym. Bull..

[75] Ye C., Miao C., Yu L., Dong Z., Zhang J., Mao Y., Lu X., Lyu Q. (2019). Factors Affecting the Efficacy and Safety of Aminophylline in Treatment of Apnea of Prematurity in Neonatal Intensive Care Unit. Pediatr. Neonatol..

[76] Ahmad W., Hassan Y.A., Ahmad A., Suroor M., Sarafroz M., Alam P., Wahab S., Salam S. (2023). A Simple Stability-Indicating UPLC Method for the Concurrent Assessment of Paracetamol and Caffeine in Pharmaceutical Formulations. Separations.

[77] Billowria K., Sandhu N.K. (2023). Degradation Pathway of Caffeine in Bulk Drug and Pharmaceutical Dosage Form. Int. J. Creat. Res. Thoughts (IJCRT).

[78] Xie F., Ji S., Cheng Z. (2015). In Vitro Dissolution Similarity Factor (F2) and in Vivo Bioequivalence Criteria, How and When Do They Match? Using a BCS Class II Drug as a Simulation Example. Eur. J. Pharm. Sci..

